# Infection-Mediated Shifts in the Microbial Communities of Deer-Fed *Ixodes scapularis* Ticks

**DOI:** 10.3390/microorganisms13112635

**Published:** 2025-11-20

**Authors:** Patil Tawidian, Bradley J. Tucker, Tela E. Zembsch, Hon S. Ip, Lyric C. Bartholomay

**Affiliations:** 1 U.S. Geological Survey, National Wildlife Health Center Madison, 6006 Schroeder Road, Madison, WI 53711, USA; tawidian@wisc.edu (P.T.); honsip@gmail.com (H.S.I.); 2 Department of Pathobiological Sciences, School of Veterinary Medicine, University of Wisconsin-Madison, 1630 Linden Drive, Madison, WI 53706, USA; zembsch@wisc.edu; 3 Department of Entomology, College of Agricultural and Life Sciences, University of Wisconsin-Madison, 1656 Linden Drive, Madison, WI 53706, USA; bjtucker2@wisc.edu

**Keywords:** *Ixodes scapularis*, blacklegged tick, *Borrelia*, microbiota, mycobiota, microbial networks

## Abstract

The holobiont of the blacklegged tick (*Ixodes scapularis*) includes maternally inherited rickettsial endosymbionts and environmentally acquired microbes that may influence tick fitness and vector competence. While previous studies have focused on characterizing the microbiota of *I. scapularis* ticks, less is known about the influence of tick infection status on microbial assemblages. Here, we collected engorged female *I. scapularis ticks* from hunter-harvested white-tailed deer (*Odocoileus virginianus*) across 11 counties in Wisconsin during fall 2022. The ticks were maintained in laboratory conditions for oviposition and then frozen for nucleic acid extraction. The infection status of each tick was determined using qPCR, targeting *Borrelia* spp., *Babesia* spp., and Powassan virus. Bacterial and fungal communities were characterized through amplicon-based sequencing targeting the 16S rRNA gene and ITS2 region, respectively. Our targeted pathogen testing revealed that 14.1% of the collected ticks were infected with *Babesia odocoilei* and 23.3% with *Borrelia burgdorferi*. The microbial community composition of ticks was significantly influenced by infection status and pathogen identity. Notably, *Borrelia*-infected ticks exhibited distinct microbiota profiles and increased microbial network connectivity. These findings provide new insights into the microbial ecology of deer-fed *I. scapularis* ticks and highlight the role of infection in shaping both microbiota and mycobiota communities.

## 1. Introduction

The blacklegged tick (*Ixodes scapularis*) is of considerable public health importance due to its ability to transmit numerous disease-causing pathogens, including *Borrelia burgdorferi*, the primary etiological agent of Lyme disease in North America [1,2]. Lyme disease remains the most frequently reported tick-borne disease (TBD) in the United States, with an estimated 476,000 individuals diagnosed and treated annually [3]. In addition to *Bo. burgdorferi*, *I. scapularis* transmits several other pathogens of public health importance, such as *Anaplasma phagocytophilum*, *Ehrlichia* spp., *Babesia* spp., and Powassan virus, all of which contribute to a growing burden of TBDs [1,2]. Beyond pathogens, *I. scapularis* ticks harbor a complex holobiont consisting of both maternally acquired endosymbionts and non-endosymbiont microbial communities [4,5]. Endosymbionts within the genus *Rickettsia*, for example, are hypothesized to play a pivotal role in *I. scapularis* development by supplementing essential nutrients [6,7,8]. However, the ecological drivers underlying non-endosymbiont microbial community assembly (a defined group of organisms that occur in space and time), as well as the functional implications of these communities for tick physiology and vector competence, remain poorly understood.

The microbiota of *I. scapularis* ticks is likely largely acquired from the environment during questing, host attachment, and feeding [5,9,10]. Studies characterizing the microbiota composition of *I. scapularis* have reported variable abundance of non-endosymbiont bacterial taxa associated with soil and plants, further supporting environmental acquisition [11,12,13,14]. The drivers of microbiota assembly are largely associated with environmental factors, such as geographic location [15,16,17], along with biological factors including ontogeny and sex [11,13,15,17,18]. Additionally, studies have revealed that *Bo. burgdorferi* infection in *I. scapularis* nymphs alters microbiota composition without significantly affecting overall community richness or diversity [12,19]. This infection-associated shift in microbial community structure has been proposed to promote *Bo. burgdorferi* infection, as dysbiosis in *I. scapularis* nymphs impairs midgut colonization by the pathogen [20,21]. However, less is known about the influence of infection status on microbial community composition and assembly in field-collected and engorged *I. scapularis* ticks.

In addition to the microbiota, relatively little is known about the mycobiota (fungal component of the holobiont) and its composition and assembly in ticks. Culture-dependent studies have identified numerous saprophytic fungi and potential entomopathogens associated with field-collected *I. scapularis* ticks [22,23,24]. However, only a limited number of studies have characterized tick-associated fungal communities using culture-independent, sequencing-based approaches. For example, fungi from *I. scapularis* questing ticks collected from Vermont were dominated by fungi of the genus *Malassezia*, a mammal-associated yeast, along with other soil- and plant-associated taxa such as *Cladosporium* sp. [12]. Similarly, the mycobiota of field-collected *Dermacentor nuttalli* ticks was primarily composed of saprophytic fungi, including *Cladosporium* sp. [25]. These findings suggest that field-collected ticks harbor a diverse mycobiota with variable richness and composition, which contributes to the tick holobiont and may influence key biological traits.

The goals of this study were to characterize the microbiota and mycobiota of *I. scapularis* adult female ticks, which were harvested from white-tailed deer (*Odocoileus virginianus*) in Wisconsin and allowed to oviposit, and assess whether tick-borne pathogens (TBPs) may influence tick holobiont composition and diversity. In this study, we collected live and engorged *I. scapularis* female ticks from deer harvested in 11 counties in Wisconsin. We used targeted pathogen testing to determine the infection status of each tick. Using amplicon-based sequencing of the 16S rRNA and Internal Transcribed Spacer 2 (ITS2) regions, we amplified their associated microbiota and mycobiota. Our results demonstrate that *I. scapularis* harbors a diverse community of non-endosymbiont bacterial taxa, and that the composition of this microbiota is influenced by tick infection with TBPs. In contrast, while fungal taxa were also detected, the mycobiota composition and diversity appeared to be less affected by infection status. These findings highlight the differential effects of pathogen presence on the bacterial and fungal components of the *I. scapularis* holobiont.

## 2. Materials and Methods

### 2.1. Tick Collection and Sample Preparation

During the late fall 2022 hunting season, engorged female *I. scapularis* ticks were collected from white-tailed deer hunter-harvested from 11 counties across Wisconsin to establish a laboratory *I. scapularis* colony. Heads from white-tailed deer hunted across all counties were brought to the deer registration station at Fort McCoy, a United States Army installation located in Monroe County, Wisconsin and inspected for ticks. Inspections for ticks consisted of combing fingers through pelage on the head and behind the ears. When detected, ticks were removed using tweezers by grasping the body as close to the skin as possible, twirling in a circular motion, and pulling upwards with steady pressure to avoid damaging the tick’s mouthparts. Upon removal, ticks from the same deer were transferred into a single 50 mL Falcon tube (Becton Dickinson and Company, Franklin Lakes, NJ, USA) containing a wet filter paper to maintain a humid environment. A total of 163 engorged female ticks were collected from 90 deer carcasses for downstream processing. The deer were hunted across 11 counties in Wisconsin. The majority of retrieved ticks were collected from deer hunted in Monroe County (n = 133), followed by Dane (n = 8), Wood (n = 3), two ticks from each of Sauk and Richland counties, and one tick from each of Clark, Columbia, Dunn, Green, La Crosse, and Marquette counties. Nine ticks were harvested from deer from unknown hunting locations. While these geographic locations provide context for where deer were hunted, they do not reflect the location of tick attachment to their host. Therefore, geographic data are provided to show the region distribution of sampled hosts and were not included in downstream analyses of microbial community composition.

Upon transfer to the laboratory, pooled ticks were separated and washed with sterile distilled water once to remove residues and loosely attached contaminants from tick surfaces and then placed in humid chambers that consisted of a food storage container (Cambro Manufacturing, Huntington Beach, CA, USA) with a sponge imbibed with water. The humidity chambers were placed in an incubator at 22 °C, 90% humidity, and a 16:8 hr Light–Dark (L:D) cycle. Ticks were not surface-sterilized upon arrival at the laboratory due to their use in subsequent colony establishment. To establish a laboratory tick colony, engorged ticks were allowed to oviposit for a period of three weeks and were then frozen at −80 °C for nucleic acid extraction. Ticks that died prior to oviposition were frozen at −80 °C upon death. Frozen ticks were then used for pathogen testing and downstream microbiota/mycobiota analysis described in this manuscript.

### 2.2. Nucleic Acid Extraction

Extractions of individual tick DNA and RNA were performed separately as previously described [26]. In brief, individual female *I. scapularis* ticks were bisected using a single-edge razor blade (AccuTec Blades, Inc., Verona, VA, USA) and placed in 1.7 mL centrifuge tubes. DNA extraction was performed using the Bioline Isolate II Genomic DNA Kit (Meridian Life Science, Inc., Memphis, TN, USA) following the manufacturer’s instructions with the minor modification of elution in 75 μL EDTA-free elution buffer. Prior to RNA extraction, bisected ticks were mechanically disrupted using a mini-Beadbeater-24 (BioSpec Products, Bartlesville, OK, USA) with one 4.5 mm stainless bead per tick. RNA extraction was performed using the Qiagen RNeasy Mini Kit (Qiagen, Germantown, MD, USA). Extracted DNA and RNA were quantified using Qubit 1X dsDNA HS Assay Kit (Thermo Fisher Scientific, Waltham, MA, USA) and Qubit RNA HS Assay Kit, respectively (Thermo Fisher Scientific, Waltham, MA, USA).

### 2.3. Pathogen Testing

Specific pathogens with a potential of transovarial transmission were detected using previously described quantitative PCR (qPCR) and reverse transcriptase PCR (RT-PCR) assays (Table 1) [26,27,28,29]. In brief, real-time fluorescent resonance energy transfer (FRET) PCR assays targeting *Bo. burgdorferi* sensu stricto and *Bo. miyamotoi* were conducted in a 20 µL total reaction volume, containing 5 µL of DNA template, 1× FastStart^TM^ PCR Mastermix (Sigma Aldrich, St. Louis, MO, USA), 500 nM each of forward and reverse primers, and 200 nM each of probes. The reaction was performed using 45 cycles of thermal cycling with an initial denaturation step at 95 °C for 10 min, followed by denaturation at 95 °C for 10 s, annealing at 55 °C for 10 s, and extension at 72 °C for 12 s.

In addition, FRET assays were used to detect *Ba. microti* and *Ba. odocoilei* in 20 µL total reaction volume containing 5 µL DNA template, 1× FastStart Enzyme, 1× Hybridization Probe, 3 mM magnesium chloride, and a commercially available primer–probe combination referred to as LCBAB1 2222 (TIB MolBiol, Adelphia, NJ, USA) [28]. The reaction was performed using 45 cycles of thermal cycling with an initial denaturation step at 95 °C for 10 min, followed by denaturation at 95 °C for 10 s, annealing at 55 °C for 15 s, and extension at 72 °C for 15 s, at a ramp rate of 20 °C/s. Each assay included a no-template control and a synthetic target gene fragment as a positive control. All assays were performed on the Roche LightCycler 2.0 or 480 instruments (Roche Diagnostics, Basel, Switzerland) and used FRET probes for the detection and identification of the amplified nucleic acid.

Powassan virus was detected using the AccessQuick RT-PCR System (Promega, Madison, WI, USA) with the following concentration per 1× reaction: 5.6 µL DNase-free water, 7.5 µL 2× reaction buffer, 0.3 µL of each primer, 0.3 µL of *Taq*, and 1 µL of template. Reverse transcription step was performed at 45 °C for 45 min. Initial denaturation step was performed at 95 °C for 2 min, followed by 30 cycles of denaturation at 95 °C for 30 s, annealing at 55 °C for 1 min, extension at 72 °C for 1 min, and a final extension step for 2 min. Successful PCR amplification was determined by visualizing 5 µL of the PCR products on 1% agarose gels.

### 2.4. Microbial Community Amplification and Sequencing

#### 2.4.1. Bacterial 16S Library Construction

Purified genomic DNA was submitted to the University of Wisconsin-Madison Biotechnology Center. The DNA concentration was verified fluorometrically using either the Qubit^®^ dsDNA HS Assay Kit or Quant-iT™ PicoGreen^®^ dsDNA Assay Kit (ThermoFisher Scientific, Waltham, MA, USA). The samples were prepared in a similar process to the one described in Illumina’s 16S Metagenomic Sequencing Library Preparation Protocol, Part # 15044223 Rev. B (Illumina Inc., San Diego, CA, USA), with the following modifications: The 16S rRNA gene V3/V4 variable region was amplified with fusion primers (forward primer 341f: 5′-ACACTCTTTCCCTACACGACGCTCTTCCGATCT(N)_0/6_CCTACGGGNGGCWGCAG-3′, reverse primer 805r: 5′-GTGACTGGAGTTCAGACGTGTGCTCTTCCGATCT(N)_0/6_GACTACHVGGGTATCTAATCC-3′). Region-specific primers were previously described in [30] and were modified to add 0 or 6 random nucleotides ((N)_0/6_) and Illumina adapter overhang nucleotide sequences to the 5′ end of the gene-specific sequences. Following initial amplification, reactions were cleaned using a 0.7x volume of AxyPrep Mag PCR clean-up beads (Axygen Biosciences, Union City, CA, USA). In a subsequent PCR, Illumina dual indexes and sequencing adapters were added using the following primers (forward primer: 5′-ATGATACGGCGACCACCGAGATCTACAC[i5 index]ACACTCTTTCCCTACACGACGCTCTTCCGATCT-3′, Reverse Primer: 5′-CAAGCAGAAGACGGCATACGAGAT[i7 index]GTGACTGGAGTTCAGACGTGTGCTCTTCCGATCT-3′, where bracketed sequences are 10 bp custom Unique Dual Indexes).

#### 2.4.2. Fungal Internal Transcribed Spacer Region Library Construction

The DNA concentration was verified fluorometrically using either the Qubit^®^ dsDNA HS Assay Kit or Quant-iT™ PicoGreen^®^ dsDNA Assay Kit (ThermoFisher Scientific, Waltham, MA, USA). Samples were prepared in a similar process to the one described in Illumina’s 16S Metagenomic Sequencing Library Preparation Protocol, Part # 15044223 Rev. B (Illumina Inc., San Diego, CA, USA) with the following modifications: The ITS region was amplified with fusion primers (forward primer: 5′-ACACTCTTTCCCTACACGACGCTCTTCCGATCTCTTGGTCATTTAGAGGAAGTAA-3′, reverse primer: 5′-GTGACTGGAGTTCAGACGTGTGCTCTTCCGATCTTCCTCCGCTTATTGATATGC-3′). Region-specific primers were previously described (ITS1-F [31]; ITS4 [32]) and were modified to add Illumina adapter overhang nucleotide sequences to the region-specific sequences. Following initial amplification, reactions were cleaned using a 0.7× volume of AxyPrep Mag PCR clean-up beads (Axygen Biosciences, Union City, CA, USA). Using the initial amplification products as template, a second PCR was performed with primers that contain Illumina dual indexes and sequencing adapters (Forward primer: 5′-AATGATACGGCGACCACCGAGATCTACAC[i5 index]ACACTCTTTCCCTACACGACGCTCTTCCGATCT-3′, Reverse Primer: 5′-CAAGCAGAAGACGGCATACGAGAT[i7 index]GTGACTGGAGTTCAGACGTGTGCTCTTCCGATCT-3′, where bracketed sequences are equivalent to the Illumina Dual Index adapters D501-D508 and D701-D712, N716, N718-N724, N726-N729).

### 2.5. Sequencing

PCR reactions were cleaned using a 0.7× volume of AxyPrep Mag PCR clean-up beads (Axygen Biosciences, Union City, CA, USA). The quality and quantity of the finished libraries were assessed using an Agilent 4200 TapeStation DNA 1000 kit (Agilent Technologies, Santa Clara, CA, USA) and Qubit^®^ dsDNA HS Assay Kit, respectively. Libraries were pooled in an equimolar fashion and appropriately diluted prior to sequencing on a single flow cell. Paired-end 2 × 150 bp reads were generated using Illumina NextSeq 2000 (Illumina Inc., San Diego, CA, USA). Extraction controls did not yield sequencing libraries, limiting our ability to detect potential low-abundance background contaminants.

### 2.6. Data Analysis

#### 2.6.1. 16S Sequence Processing

Paired-end sequences (25,251,352 raw reads) from 163 individual *I. scapularis* ticks were processed using mothur (v.1.48.2) [33] (Text S1). Data denoising included filtering out 3,309,875 short and ambiguous reads and homopolymers longer than 8 bp. High-quality sequence reads were aligned to the SILVA ribosomal RNA database [34] and clustered, allowing up to two nucleotide differences. In addition, 185,556 chimeric sequences were removed using VSEARCH v2.28.1 [35]. Sequence reads were either unclassified or classified as Plantae, Archaea, or Metazoa (Table S1). The remaining dataset consisted of 623,567 unique reads and a total of 21,755,921 high-quality paired-end reads. Unique sequences were then classified to Amplicon Sequence Variants (ASVs) using the SILVA ribosomal RNA database. Low-abundance ASVs of less than ten reads were filtered out, resulting in a final dataset that consisted of 4798 ASVs and 21,066,238 sequence reads (Table S2). Rarefaction curves across tick samples were mostly saturated, indicating sufficient sequencing depth to capture the bacterial community (Figure S1).

Upon the completion of data processing, we generated two datasets to compare the community diversity in the presence and absence of the *I. scapularis* endosymbiont *Rickettsia tamurae* subsp. *buchneri*. The first dataset, referred to as “Endosymbiont+”, included *R. tamurae* subsp. *buchneri* ASVs and reads and was subsampled to 19,251 reads, resulting in a dataset with 2,854,216 reads and 3458 ASVs. The second dataset, referred to as “Endosymbiont–”, excluded *R. tamurae* subsp. *buchneri* ASVs and reads and was subsampled to 1035 reads. This resulted in a dataset having 1478 ASVs that accounted for 143,865 reads across 140 individual *I. scapularis* tick samples. The different rarefaction depths reflect the distinct sequencing depth of each dataset, and each was analyzed independently to explore within-group diversity and composition. The Good’s coverage across both datasets was near completion (0.999 ± 0.0003 standard deviation).

#### 2.6.2. ITS Sequence Processing

Paired-end ITS sequences (4,749,166 raw reads) from 163 individual *I. scapularis* ticks were processed using mothur (v.1.48.2) [33] (Text S2). The removal of ambiguous reads, homopolymers longer than 10 bp, and chimeric sequences resulted in a dataset that consisted of 576,566 sequence reads. Fungal sequences were assigned to taxa using the naive Bayesian classifier against the UNITE-curated International Nucleotide Sequence Database reference database [36]. Fungal sequences were clustered into Operational Taxonomic Units (OTUs) using the average neighbor algorithm (unweighted pair group method using average linkages [UPGMA]) at a 97% similarity threshold. In addition, OTUs with fewer than ten reads were removed from the dataset. Rarefaction analysis showed variable sequencing depth across the samples, with some reaching saturation while others did not (Figure S2). Notably, several samples exhibited sequencing issues, including high levels of homopolymers and extremely low read counts, likely due to poor amplification or sequencing artifacts. These samples were excluded from downstream analysis to avoid introducing noise and artificial diversity. To reduce sequencing bias, the dataset was subsampled to 1000 reads per individual tick. This resulted in a filtered dataset consisting of 359 OTUs and 34,000 reads across 34 ticks (Table S3). The Good’s coverage of the 34 tick samples remaining was high, indicating reliable mycobiota characterization (0.953 ± 0.045 standard deviation).

#### 2.6.3. Community and Network Analyses

To determine bacterial and fungal community richness and diversity across tick samples, observed richness (S*_obs_*) and Shannon’s diversity index (H’) were estimated using mothur (v.1.48.2). Each alpha diversity metric was checked for normality using the Shapiro–Wilk test. To assess whether alpha diversity indices varied across tick samples based on infection status, we performed a non-parametric Kruskal–Wallis test. Due to a low sample size (n = 1), tick samples co-infected with *Borrelia* sp. and *Babesia* sp. were excluded from the statistical analysis. To assess microbial community composition changes across infection status, we calculated the Bray–Curtis dissimilarities across tick samples for the bacterial and fungal communities separately. We then visualized the observed dissimilarities using Principal Coordinate Analysis (PCoA). We assessed significant differences in community composition using PERMANOVA across infection status and pathogen detected. Statistical analyses were performed using the stats package in the R statistical computing software (v.2024.12.1) [37]. In addition to subsampled analyses, we performed a centered log-ratio (CLR) transformation of count data to account for the compositional nature of sequencing data. Euclidean distances were then calculated on CLR-transformed values, and PERMANOVA was applied as above. Differential abundance analysis was conducted using LEfSe (Linear Discriminant Analysis Effect Size) (v.1.1.2) on the Galaxy platform, employing a Kruskal–Wallis alpha of 0.05 and an LDA score (log_10_) threshold of 2.0 to determine significantly enriched taxa [38].

Finally, to determine the association of tick infection with *Borrelia* sp. on tick microbiota, network comparisons between infected and uninfected ticks were performed on the raw dataset (i.e., not subsampled) using the NetCoMi R package (v1.2.0) [39]. Prior to network construction, all *Borrelia* ASVs were removed from infected samples to avoid biasing the network, since infection status (presence/absence of *Borrelia*) defines the grouping. Including *Borrelia* would dominate the data and distort associations among other bacteria. ASVs were then combined to the genus level and used in network construction. Differential networks were constructed based on the Semi-Parametric Rank-based approach for INference in Graphical model (SPRING) using the “netConstruct” function with a 0.05 threshold, layout method as “approx”, and 500 iterations to ensure network stability. As sparsification, normalization, and zero handling are performed internally in SPRING, “normMethod” and “zeroMethod” were assigned as “none”. Internally, SPRING applies a modified centered log-ratio (mCLR) transformation to normalize compositional microbiota data while accommodating zero values. Additionally, SPRING uses a semi-parametric, rank-based estimation approach based on a truncated Gaussian copula model, which is designed to handle the zero datasets without requiring external preprocessing. Since the uninfected group contained a higher number of samples, relying on a single selection based on taxa frequency could introduce sampling bias. To mitigate this, the dataset was randomly subsampled 50 times, and differential networks were reconstructed for each subset. While individual networks are not reported, bar plots summarizing key topological metrics, including modularity, natural connectivity, and clustering coefficient, across the 50 iterations were used to assess the stability and robustness of the uninfected dataset network structure. Network analysis was then performed using the “netAnalyze” function of the NetCoMi package using the “cluster_fast_greedy” algorithm and computing normalized betweenness and degree for the whole network and the largest connected component (LCC) of the network. Network comparisons were performed using the “netCompare” function with adaptive Benjamin–Hochberg multiple testing and a quantile threshold of 0.85 for Jaccard’s index at 1000 permutations.

To evaluate the network topology, we analyzed (1) the whole network modularity—a measure of the degree to which a network can be divided into distinct, densely connected subgroups (or modules); (2) the whole network positive edge percentage—the proportion of edges in a network that represent positive interactions between nodes, relative to the total number of edges; (3) the whole network edge density—a measure of network connectivity; (4) the relative largest connected component (LCC) size—the largest subset of nodes (in this case genera) in the network that are all directly or indirectly connected; and (5) the average path length of LCC—a measure of distance between nodes.

## 3. Results

### 3.1. Natural Infection of Ticks Harvested from Deer in Wisconsin

Of the 163 ticks harvested from white-tailed deer, a total of 44.2% (n = 72) were infected with at least one pathogen. Among the *Borrelia* species tested by qPCR, *Bo. burgdorferi* was the predominant species, detected at 23.3% (n = 38), while *Bo. miyamotoi* was found in 4.29% (n = 7) of tick samples (Table 2 and Table S4). One tick was co-infected with both *Borrelia* species. *Borrelia*-positive ticks were harvested from deer hunted in six counties, including Monroe (n = 27), followed by two positive ticks from Dane County and one positive tick in each of Columbia, Dunn, La Crosse, and Sauk counties. Of the 44 *Borrelia*-positive tick samples, five ticks were assigned as “unknown” due to missing county information. The majority (95%) of the ticks that were identified as *Borrelia*-positive with qPCR were also positive using NGS.

The majority of *Babesia*-positive tick samples were infected with *Ba. odocoilei* (n = 24), and one tick sample was positive for *Ba. microti* (Table 2 and Table S4). *Babesia*-positive tick samples were identified in ticks collected from three counties, Monroe (n = 18), Dane (n = 2), and Richland (n = 1). Four ticks were assigned as “unknown” due to a lack of county information. Of the 163 ticks sampled, one tick was positive for POWV (Table 2 and Table S4). Upon subsampling, the dataset consisted of 18 *Babesia*-positive ticks, 38 *Borrelia*-positive ticks, and 84 uninfected ticks.

### 3.2. Microbial Community Composition of Deer-Fed Ixodes Scapularis Ticks

#### 3.2.1. Microbiota Composition of Ixodes Scapularis Ticks

**Figure 1:**
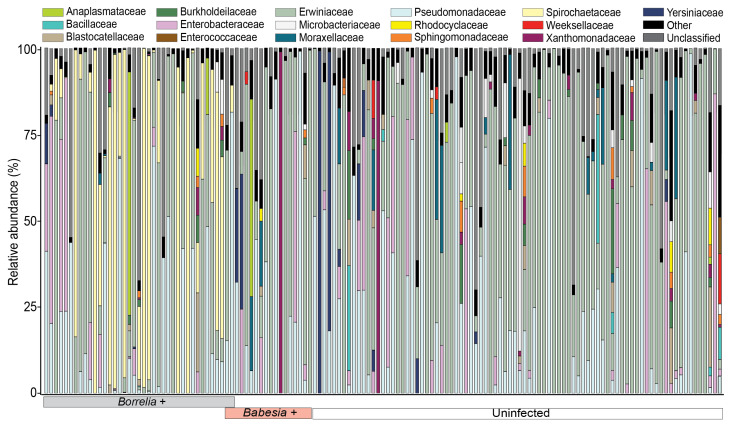
Bar plot of the relative abundances of bacterial families in the absence of endosymbiont reads across *Ixodes scapularis* ticks collected from white-tailed deer. The relative abundance of bacterial families associated with individual tick samples is arranged based on infection status and pathogen. Bar colors correspond to bacterial families as indicated in the legend above the plot. Tick infection status is indicated by colored boxes under the x-axis. Low-abundance families that account for less than 10% of sequence reads per sample are combined and categorized as “Other”. Reads from Amplicon Sequence Variants (ASVs) that were not assigned to the family level were combined and categorized as “Unclassified”.

In the endosymbiont-positive dataset, *R. tamurae* subsp. *buchneri* accounted for most sequencing reads (15,679,185 reads; 74.4%) associated with individual *I. scapularis* ticks, followed by reads belonging to the phylum Proteobacteria (Figure S3). To characterize the non-endosymbiont microbiota composition of individual ticks, we plotted the relative abundance of bacterial ASVs at the family level in the absence of endosymbiont reads (Figure 1). The phylum Proteobacteria was dominant across tick samples and accounted for more than 90% of total sequence reads, irrespective of pathogen and infection status (Table S2). The remaining low-abundance phyla, which accounted for 1–2% of sequence reads across all tick samples, were Actinobacteriota, Acidobacteriota, and Firmicutes (Table S2). Within the phylum Proteobacteria, similar families were detected across tick samples, irrespective of infection status. However, the relative abundance of sequence reads across the detected families varied depending on infection status and pathogen detected (Figure 1).

The family Erwiniaceae was dominant across most tick samples, accounting for 41.6%, 50.1%, and 56.1% of reads in *Babesia*-positive, uninfected, and *Borrelia*-positive ticks, respectively. The second most common family was Pseudomonaceae. *Babesia*-infected ticks had the highest relative abundance (29.9%), as compared to *Borrelia*-positive (16.7%) and uninfected ticks (19.2%), respectively (Figure 1). Similarly, *Babesia*-positive samples had the highest relative abundance of taxa within the Xanthomonadaceae (6.7%) and Moraxellaceae (5.8%) families compared to uninfected ticks (2.3% and 3.5%, respectively) and *Borrelia*-positive ticks (2.8% and 0.500%, respectively). In contrast, *Borrelia*-positive ticks had the highest relative abundance of Enterobacteriaceae (11.4%) compared to uninfected (8.1%) and *Babesia*-positive ticks (4.9%) (Figure 1).

#### 3.2.2. Mycobiota Composition of Ixodes Scapularis Ticks

Of the 163 tick samples sequenced, the majority of ticks had low fungal richness and diversity and were excluded from downstream analysis (Figure S2). The final dataset consisted of 10 ticks that were *Borrelia*-positive compared to 24 ticks that were uninfected. In uninfected ticks, the phylum Ascomycota accounted for 89.1% of sequence reads, with the most dominant order being Cladosporiales (family Cladosporiaceae) (89.2%) by Hypocreales (family Nectriaceae) (5.1%), Pleosporales (families Pleosporaceae, Phaeosphaeriaceae, and Didymellaceae) (3.0%), and Eurotiales (family Aspergillaceae) (2.1%). The remaining 0.6% ascomycetous reads were associated with low-abundance orders. Similarly, the phylum Ascomycota was the most abundant in *Borrelia*-positive ticks (66.3%). In addition, the order Cladosporiales remained the dominant order in *Borrelia*-positive ticks. However, its relative abundance was lower (68.1%) compared to uninfected ticks. The order Eurotiales was the second dominant order (23.6%) in infected ticks, followed by Pleosporales (7.1%) (Figure S4). The remaining 1.2% of sequence reads were assigned to low-abundance ascomycetous orders.

The abundance of the Basidiomycota phylum varied depending on tick infection status. *Borrelia*-infected ticks had a higher relative abundance of basidiomycetous fungi (33.7%) as compared to uninfected ticks (10.9%). The basidiomycetous order Tremellales (families Bulleribasidiaceae and Rhynchogastremaceae) accounted for 31.8% of sequence reads compared to 10.9% in uninfected ticks, contributing to the observed shift in relative abundance of basidiomycetes in infected ticks (Figure S4). In addition, the order Trichosporonales (family Trichosporonaceae) accounted for 1.9% of sequence reads in infected ticks and was not identified in uninfected ticks.

### 3.3. Shift in Relative Abundance of Bacterial and Fungal Genera in Response to Infection

**Figure 2:**
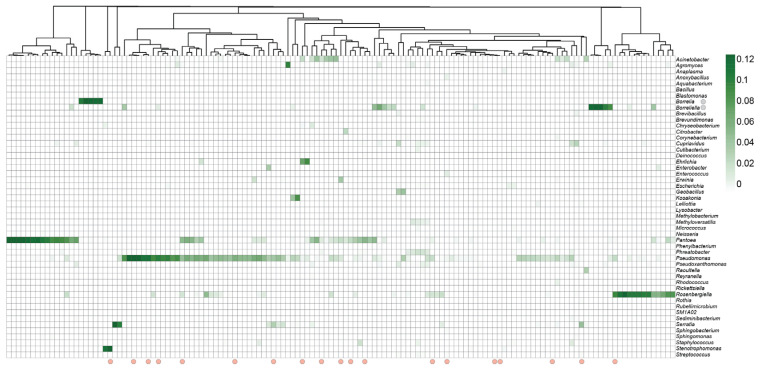
Heatmap of the relative abundances of the top 50 bacterial genera identified across *Ixodes scapularis* ticks. Heatmap showing hierarchical clustering of tick samples based on Euclidean distance of Amplicon Sequence Variant (ASV) relative abundances. The y-axis displays the top 50 most abundant bacterial genera across all samples. The x-axis represents individual tick samples. Pink circles at the bottom of the x-axis indicate samples that tested positive for *Babesia* spp. (n = 18). Gray circles at the y-axis highlight *Borrelia* spp. (n = 38) detected across individual ticks.

While major bacterial families within the phylum Proteobacteria were identified across most tick samples, the relative abundance of specific genera within this family varied according to infection status. The genus *Pseudomonas* was identified in more than 60% of tick samples (n = 85), regardless of infection status, and accounted for more than 26.4%, 24.5%, and 40.8% of sequence reads of uninfected and *Borrelia*- and *Babesia*-infected ticks, respectively (Figure 2). Several other taxa had varied relative abundances based on infection status. For example, the genus *Pantoea* was more abundant in uninfected ticks (25.1%) compared to *Borrelia*- and *Babesia*-infected ticks (14.6% and 14.1%, respectively). Similarly, the genus *Rosenbergiella* accounted for 14.5% sequence reads in uninfected ticks as compared to 6.7% and 9.1% in *Borrelia*- and *Babesia*-infected ticks, respectively. Conversely, some genera varied in relative abundance according to pathogens detected. Notably, among infected ticks, the genus *Acinetobacter* was only detected in *Babesia*-infected ticks (7.8%) and at a higher abundance than in uninfected ticks (4.9%) (Figure 2).

Similarly, shifts in the abundance of some fungal species were observed in *Borrelia*-infected and uninfected ticks (Figure S5). *Cladosporium pseudocladosporioides* was the most common ascomycetous species in uninfected ticks, accounting for 74.6% of reads, followed by *Cladosporium halotolerans* (8.0%), *Vishniacozyma carnescens* (5.2%), *Fusarium temperatum* (4.6%), *Vishniacozyma foliicola* (3.6%), and *Papiliotrema flavescens* (1.2%) (Figure S5). The remaining 2.9% of reads were associated with low-abundance fungal species. In contrast, *Borrelia*-infected ticks had a significantly lower abundance of *C. pseudocladosporioides* (43.2%) (5.55 LDA, *p* = 0.049) compared to uninfected ticks. In addition, several ascomycetous and basidiomycetous species were more abundant in *Borrelia*-infected ticks compared to uninfected ticks. For example, *Penicillium citrinum* accounted for 15.7% of sequence reads compared to 0.35% in uninfected ticks. Similarly, the basidiomycetous yeasts belonging to the genera *Vishniacozyma* and *Hannaella* accounted for 21.2% and 6.8% of sequence reads in infected ticks compared to 8.8% and 0.62% in uninfected ticks, respectively (Figure S5).

### 3.4. Bacterial Community Composition and Diversity Differ Between Infected and Uninfected Ticks

**Figure 3:**
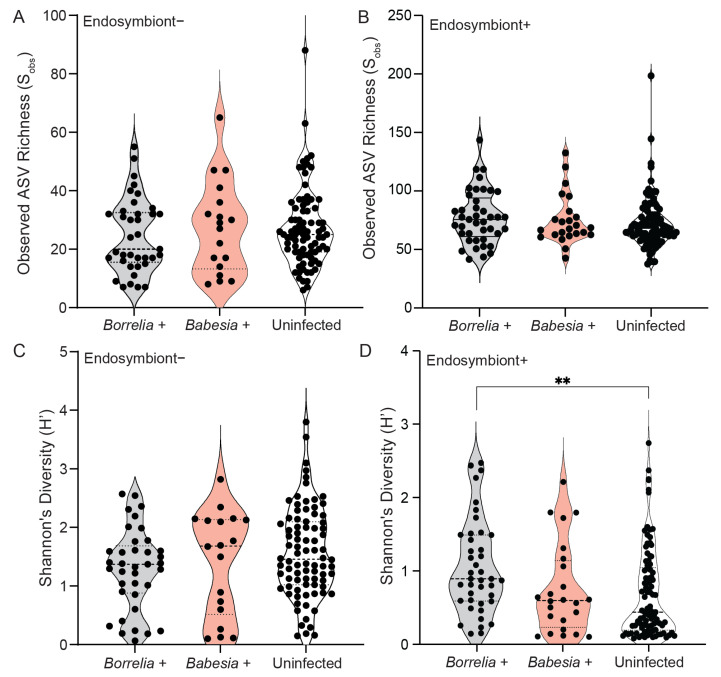
Alpha diversity indices as measured by observed Amplicon Sequence Variant (ASV) richness (S_obs_) and Shannon’s diversity (H’) of infected and uninfected *Ixodes scapularis* samples. (**A**) Observed Amplicon Sequence Variant (ASV) richness (S_obs_) across *Borrelia*-positive (n = 38), *Babesia*-positive (n = 18), and uninfected ticks (n = 84) in the absence of endosymbiont reads. (**B**) Observed Amplicon Sequence Variant (ASV) richness (S_obs_) across *Borrelia*-positive (n = 44), *Babesia*-positive (n = 24), and uninfected ticks (n = 95) in the presence of endosymbiont reads. (**C**) Shannon’s diversity across (H’) across *Borrelia*-positive (n = 38), *Babesia*-positive (n = 18), and uninfected ticks (n = 84) in the absence of endosymbiont reads. (**D**) Shannon’s diversity (H’) across *Borrelia*-positive (n = 44), *Babesia*-positive (n = 24), and uninfected ticks (n = 95) in the presence of endosymbiont reads. Statistical significance was determined using the Kruskal–Wallis test. *p* < 0.05 was considered significant, and ** denotes *p* < 0.01.

To determine whether the infection status of *I. scapularis* ticks affects bacterial species richness and diversity, we analyzed the observed ASV richness (S_obs_) and Shannon’s diversity (H’) indices (Figure 3). Our results revealed low to moderate bacterial richness (27 ± 15.1 SD) and diversity (1.5 ± 0.766) across tick samples in the absence of endosymbiont reads (Figure 3A,B). In addition, no significant differences in ASV richness and diversity were observed, regardless of infection status and pathogen detected. In the presence of endosymbiont reads, richness did not vary across infection status (Figure 3C). However, bacterial diversity was significantly higher in *Borrelia*-positive ticks (1.05 ± 0.683 SD) compared to *Babesia*-positive (0.750 ± 0.616 SD) and uninfected ticks (0.693 ± 0.608 SD) (Figure 3D). Fungal OTU richness (16.3 ± 11.7 SD) and diversity (0.573 ± 0.359 SD) were low across tick samples, with no significant differences observed across *Borrelia* infection status (Figure S6).

**Figure 4:**
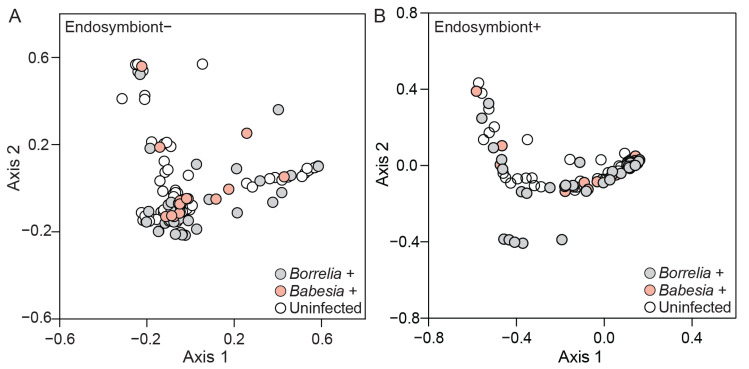
Principal Coordinate Analysis (PCoA) of Bray–Curtis dissimilarity distances across *Borrelia*- and *Babesia*-infected and uninfected *Ixodes scapularis* ticks. (**A**) PCoA of Bray–Curtis dissimilarities across axes 1 and 2 in the absence of endosymbiont reads for *Borrelia*-positive (n = 38), *Babesia*-positive (n = 18), and uninfected (n = 84) ticks. (**B**) PCoA of Bray–Curtis dissimilarities across axes 1 and 2 in the presence of endosymbiont reads for *Borrelia*-positive (n = 44), *Babesia*-positive (n = 24), and uninfected (n = 95) ticks. The keys at the bottom left of each plot indicate the color designated to tick infection status and pathogen detected.

While bacterial diversity indices did not vary across infection status of ticks, overall bacterial community composition in infected ticks varied more than in uninfected ticks. Our community analysis results revealed significant differences in bacterial community composition across infection status (Figure 4). In addition, we show that bacterial communities are altered in response to the pathogen detected. *Borrelia*-positive ticks had a significantly different bacterial community as compared to uninfected ticks, regardless of the presence or absence of *R. tamurae* subsp. *buchneri* (F = 4.15, *p* = 0.0039 and F = 1.78, *p* = 0.007, respectively) (Figure 4A,B). In contrast, the bacterial communities of *Babesia*-infected ticks did not significantly differ from those in uninfected ticks (F = 0.630, *p* = 1.00 and F = 1.07, *p* = 0.896) and *Borrelia*-positive ticks (F = 1.71, *p* = 0.230 and F = 1.36, *p* = 0.078), regardless of endosymbiont read presence or absence (Figure 4A,B). Using CLR-transformed data to account for compositional effects, *Borrelia*-positive ticks still differed significantly from uninfected ticks (F = 1.45, *p* = 0.025) and were also significantly different from *Babesia*-positive ticks (F = 2.54, *p* = 0.0003), regardless of endosymbiont presence or absence. *Babesia*-positive ticks were significantly different from uninfected ticks (F = 3.78, *p* = 0.0003). These CLR-based results confirm and slightly extend the subsampled analysis, highlighting additional separation of *Borrelia*-positive and *Babesia*-positive microbial communities. Fungal community composition did not differ across *Borrelia* infection status as compared to uninfected ticks (F = 2.07, *p* = 0.065).

### 3.5. Borrelia Infection Alters the Bacterial Network Structure in Ixodes Scapularis Ticks

**Figure 5:**
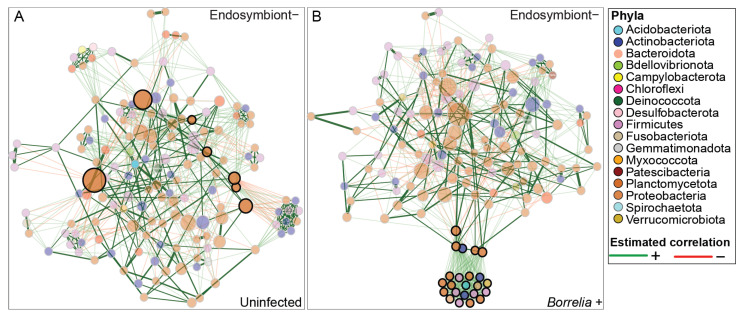
Genus-level bacterial associations across *Borrelia*-infected and uninfected ticks using the Semi-Parametric Rank-based approach for INference in Graphical model (SPRING) as an association measure. (**A**) Complete network for the uninfected (n = 84) ticks with 235 genera. (**B**) Complete network for the *Borrelia*-infected (n = 38) ticks with 235 bacteria genera. “Signed” distance metric is used to transform estimated partial correlations into dissimilarities, and non-negative similarities are used as edge weights. Red edges correspond to negative associations, while green edges correspond to positive associations. Eigenvector centrality is used for defining hubs and scaling node sizes. Hubs are defined by bold borders. Nodes (circles) are colored based on the phyla to which the identified genera belong. The key at the top right of the figure represents the color coding of each phylum and the color of the estimated associations.

To further investigate the influence of *I. scapularis* infection status on the interaction among bacterial taxa in the absence and presence of endosymbiont reads, we performed a genus-level differential network analysis of infected and uninfected ticks, using the SPRING association matrix. In the absence of endosymbiont reads, the resulting networks revealed modest differences in topological features between groups (Figure 5 and Figure S8A). Uninfected ticks exhibited slightly higher modularity (0.4539 ± 0.065) compared to infected ticks (0.454 ± 0.000), while infected ticks showed a marginally higher natural connectivity (0.011 ± 0.000 vs. 0.009 ± 0.003), indicating slightly greater overall robustness. Additionally, uninfected ticks had a higher clustering coefficient (0.403 ± 0.005) than infected ticks (0.357 ± 0.000), suggesting more localized connectivity among taxa (Figure 5A,B). However, none of these differences were statistically significant, and should therefore be interpreted as descriptive trends rather than conclusive effects of infection status. The presence of endosymbiont reads contributed to a more complex network structure in both infected and uninfected ticks, with higher modularity, natural connectivity, and clustering coefficient in the infected group, indicating a more structured and interconnected microbial network. (Figures S7 and S8B).

We observed limited overlap in the most central taxa across networks based on degree, betweenness, closeness, and eigenvector centrality. However, due to high sparsity in the full dataset and the resulting instability of permutation-based testing, we restricted statistical comparisons to a filtered dataset containing high-abundance taxa.

**Figure 6:**
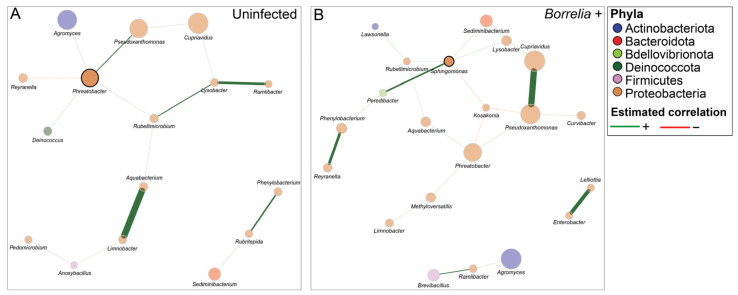
Genus-level bacterial associations using Semi-Parametric Rank-based approach for INference in Graphical model (SPRING) association measures for the top 10% of genera identified in *Borrelia*-infected (n = 38) and uninfected (n = 84) ticks. “Signed” distance metric is used to transform estimated partial correlations into dissimilarities, and non-negative similarities are used as edge weights. Red edges correspond to negative associations, while green edges correspond to positive associations. Eigenvector centrality is used for defining hubs and scaling node sizes. Hubs are defined by bold borders. Nodes (circles) are colored based on the phyla to which the identified genera belong. The key at the top right of the figure represents the color coding of each phylum and the color of the estimated associations. (**A**) Complete network for the uninfected ticks with 47 genera. (**B**) Complete network for the *Borrelia*-infected ticks with 47 genera.

To investigate the effect of abundant bacterial taxa on overall bacterial community structure across infection status, we performed a differential network analysis on taxa that occurred in more than 10% of *Borrelia*-positive samples, both with and without endosymbiont reads (Figure 6). When endosymbiont reads were included, a network was only successfully constructed for *Borrelia*-positive ticks, while no network formed for the *Borrelia*-negative group.

In the absence of endosymbiont reads, permutation-based comparison revealed no statistical significance between networks in terms of centrality-based Jaccard indices, or global graphlet correlation distance (Figure 6). However, the community structure, assessed by the Adjusted Rand Index (ARI), showed a statistically significant difference between *Borrelia*-positive and uninfected ticks (ARI = 0.315, *p =* 0.003). This result indicates a moderate level of similarity, suggesting that the grouping of taxa into modules differs between the two networks. In addition, while no significant difference in hub taxa was identified across both datasets, some taxa were influential within their respective networks. The uninfected network was characterized by the genera *Rubellimicrobium*, *Aquabacterium*, and *Limnobacter*, which exhibited high betweenness centrality (Figure 6A). In contrast, in the *Borrelia*-infected network, the genus *Sphingomonas* acted as a hub and had the highest degree, betweenness, and closeness centrality. Other genera, such as *Kosakonia*, *Peredibacter*, and *Methyloversatilis*, were also identified as key genera in *Borrelia*-infected ticks (Figure 6B).

## 4. Discussion

This study reports the microbiota/mycobiota composition of deer-fed *I. scapularis* female ticks collected from Wisconsin during the 2022 hunter-harvest season, noting that prior larval and nymphal feeding on other mammalian hosts may have contributed to their microbial communities. Our results reveal that *I. scapularis* ticks harbor a microbial community whose composition is associated with tick infection status by TBPs. We show that *Borrelia* infection in ticks is associated with shifts in microbiota structure and connectivity, characterized by a tightly interconnected community with more positive interactions. While these patterns are evident in community and network analyses, it remains unclear whether *Borrelia* infection drives the observed shifts or whether they are shaped by the microbiota during infection. In addition, while overall mycobiota richness and diversity was low in *I. scapularis* ticks, fungal community composition varied with tick infection status, with basidiomycetous fungal yeasts having a higher relative abundance in *Borrelia*-infected ticks. However, comprehensive studies are needed to draw clear conclusions about the preliminary patterns observed in the fungal community shifts reported in this study.

Our pathogen screening revealed numerous pathogens in *I. scapularis* ticks. Not surprisingly, the most detected bacterial pathogen was *Bo. burgdorferi* (23.3%), corroborating its reported prevalence in *I. scapularis* ticks in Wisconsin [40,41,42]. In addition, we identified *Bo. miyamotoi* in 4% of ticks tested across several counties. Two of these ticks were coinfected with *Bo. burgdorferi*, consistent with the report on *I. scapularis* nymph coinfection with both pathogens [42]. *Borrelia miyamotoi* was first detected in *I. scapularis* nymphs collected from several northeastern states of the United States [43]. Since its detection, it has been reported in numerous *Ixodes* sp., including *Ixodes pacificus* [44]. The prevalence of *Bo. miyamotoi* in the United States varies widely [44]. In Wisconsin the prevalence of *Bo. miyamotoi* in *I. scapularis* adults ranges between 0.63 and 1.22% [45]. In addition to bacterial pathogens, our screening detected *Babesia* sp. in 15.3% of tested ticks. However, most positive ticks were associated with *Ba. odocoilei* (14.7%). This agreed with previous surveys of *Babesia* sp. prevalence in *I. scapularis* nymphs in Wisconsin [42] and is consistent with the role of white-tailed deer as a reservoir host for *Ba. odocoilei* [46]. Finally, one tick tested positive for Powassan virus, consistent with previous reports of detection in *I. scapularis* ticks [47].

The overall goal of this study was to characterize the microbial community composition of *I. scapularis* ticks and determine the influence of natural infection on microbial community dynamics. In *I. scapularis*, *Rickettsia* sp. is reported as a key endosymbiont involved in the production of nutrients essential for tick development [5,10]. The microbiota characterization of ticks, including *I. scapularis,* is challenged by a large proportion of read assignment to the *Rickettsia* endosymbiont. Indeed, our analysis revealed that most sequencing reads were assigned to *R. tamurae* subsp. *buchneri*. Upon the depletion of these reads, our results showed the dominance of taxa within the phylum Proteobacteria (Pseudomonadota) across all tick samples, irrespective of infection status. This pattern of dominance is consistent with previous reports describing the microbiota composition of engorged and unfed *I. scapularis* ticks [11,12,14,16,48,49]. The dominance of the phylum Proteobacteria is attributed largely to the abundance of genera within the families Enterobacteriaceae and Pseudomonadaceae. Bacteria within these families have a broad ecological niche, are typically found in soil, and are associated with numerous invertebrate and vertebrate hosts [50,51]. For example, the genera *Pseudomonas* and *Pantoea* were most abundant and prevalent in our dataset. Both genera were identified using culture-dependent and sequencing strategies in several *Dermacentor* and *Ixodes* tick species [52,53,54]. Given that ticks are obligate blood-feeders, the frequent reports of soil- and plant-associated genera suggest environmental acquisition during questing or uptake during feeding through contact with host skin.

While several studies have assessed the microbiota composition of *I. scapularis* ticks, less is known about the mycobiota composition and diversity in questing and engorged ticks. Our fungal sequencing analysis identified two fungal phyla associated with *I. scapularis* ticks. The phylum Ascomycota was the most dominant, followed by Basidiomycota. This pattern of phylum dominance is reported in several insect mycobiota characterization studies and is attributed to the ubiquity of taxa within these phyla [55,56] and the primer bias during library amplification [57]. The ascomycetous genus *Cladosporium*, was the most abundant genus across *I. scapularis* ticks in our dataset. This genus consists of species that have a wide ecological range and are associated with diverse habitats, including soil, plants, and decaying organic matter [58]. Their abundance in *I. scapularis* ticks is expected due to their ubiquity in tick environments. Indeed, *Cladosporium* species have been isolated from field-collected *Ixodes frontalis* and *I. scapularis* ticks [24,53]. Similarly, the next-generation sequencing of *D. nuttalli* mycobiota revealed *Cladosporium* as the most abundant genus across tick samples [25]. Most *Cladosporium* sp. are saprophytic; however, some species exhibit entomopathogenic potential [59]. Under laboratory conditions, *Cladosporium cladosporioides* causes mortality in several insects, including aphids, mosquitoes, whiteflies, and beetles [59]. In our dataset, *C. pseudocladosporioides*, a closely related species to *C. cladoporioides*, was detected across most ticks. While the entomopathogenic potential of this species is unknown, it has been isolated from *Drosophila suzukii* larvae [60]. In addition, known entomopathogenic fungi, *Penicillium citrinum* and *Beauveria pseudobassiana* were identified in our dataset across tick samples. Both fungi have been previously isolated from ticks. Under laboratory conditions, *P. citrinum* has a reported entomopathogenicity towards *Amblyomma americanum* ticks [61]. In addition, *B. pseudobassiana* has been isolated from *I. ricinus* ticks in Moldova [62,63].

Some studies assessing the microbiota dynamics of *I. scapularis* ticks revealed that life stage [13,15,18], sex [11,17], feeding status [11,15], and collection site [15,16,17] are the major drivers of bacterial diversity in ticks. Others have reported changes in overall *I. scapularis* microbiota composition in response to *Bo. burgdorferi* infection [12,14,19]. However, less is reported on the effect of other TBPs on microbiota diversity. The disparity in the reported contribution of tick infection status to the overall microbiota composition and diversity can be attributed to several factors, including tick host preference and blood meal source [64] and the choice of bioinformatic analysis pertaining to the inclusion of endosymbiont reads, which can inflate observed richness and reduce diversity metrics [19]. Our results revealed that *I. scapularis* infection status by TBPs influences the bacterial and fungal community composition but not richness. This was largely driven by shifts in the relative abundance of some bacterial and fungal taxa across infection status and pathogen detected. For example, *Acinetobacter* was more abundant in *Babesia*-positive ticks as compared to *Borrelia*-positive and uninfected ticks. Indeed, studies assessing the association of *Haemaphysalis longicornis* adult ticks with *Ba. microti* reveal that susceptibility to infection was largely dependent on *Acinetobacter* and *Coxiella* species within the resident microbiota [65,66]. We acknowledge that distinguishing the contributions of prior blood feeding and environmental exposures during the nymphal stage from those of *Borrelia* infection status remains challenging. Nevertheless, the microbiota differences observed here are associated with infection in adult ticks collected at the same life stage and feeding status. Future controlled studies across life stage and infection status could help further elucidate the contribution of these factors to infection-associated microbiota patterns.

Similarly, differences in the relative abundance of fungal taxa were observed across uninfected and *Borrelia*-infected ticks. *C. pseudocladosporioides* was significantly less abundant in *Borrelia*-infected ticks. In addition, several yeast species within the genus *Vishniacozyma* (formerly *Cryptococcus*) had a higher relative abundance in *Borrelia*-positive ticks. The influence of these fungal taxa on tick microbiota/mycobiota interaction during infection remains largely unclear. Future studies characterizing the microbiota and mycobiota of *I. scapularis* ticks could determine the effect of key bacterial and fungal taxa on tick infection by TBPs.

Finally, our network analysis revealed that *Borrelia*-positive ticks had a modestly more connected bacterial network with positive interactions as compared to the uninfected ticks, regardless of endosymbiont read depletion. These results suggest that the infection of *I. scapularis* ticks with *Bo. burgdorferi* may positively alter the microbiota interactions to potentially enhance tick colonization. Our results are supported by previous studies showing that alteration of *I. scapularis* nymphal microbiota through antibiotic treatment and biofilm disruption reduces *Bo. burgdorferi* colonization in the tick midgut, despite successful feeding on infected mice [21,67], suggesting a role for the microbiota in facilitating *Borrelia* infection. Interestingly, network formation of abundant taxa was only observed in *Borrelia*-positive samples when endosymbiont reads were included, which may suggest increased microbial connectivity in these ticks. However, future experimental validation would be necessary to determine whether endosymbionts play a role in structuring microbial communities in a way that influences pathogen acquisition and colonization.

In addition, our network analysis of abundant taxa identified in more than 10% of ticks revealed several hub taxa, including *Sphingomonas*, that were present in the *Borrelia*-positive ticks. *Sphigonomoas* was identified as more abundant in *Bo. Burgdorferi*-positive *I. scapularis* nymphs collected in Vermont as compared to uninfected ticks using a two-way Similarity Percentages (SIMPER) analysis [12]. Thus, our results support the notion that *Bo. burgdorferi* infection in ticks alters microbiota interaction and dynamics, which ultimately may affect tick infection status. Future studies may consider investigating the role of tick holobiont in susceptibility of ticks to TBPs and assessing the contribution of tick endosymbionts in shaping these interactions.

In conclusion, this study provides a fundamental insight into the microbial community composition of deer-fed *I. scapularis* ticks and the effect of natural infection on non-endosymbiont community diversity. We show that the infection status of ticks drives the microbiota/mycobiota composition. In addition, we show that *Borrelia* infection influences tick microbiota by increasing positive interactions among resident taxa. However, determining additional environmental drivers of tick holobiont assembly, including geographic location, is crucial to better understanding microbial community dynamics. While ticks used in this study were harvested from white-tailed deer across several counties, the majority were associated with deer hunted in Monroe County, and their prior geographic origin remains unknown. Thus, future studies could perform broader geographic sampling to determine the contribution of environmental exposure to holobiont assembly and to assess the microbiota/mycobiota dynamics in response to infection by different pathogens, including *Babesia* species.

## Figures and Tables

**Table 1:** Target regions and primer/probe sequences for pathogen detection in deer-harvested ticks. Quantitative PCR (qPCR) was used for most assays; POWV was detected using conventional RT-PCR. Fluorescent probe labels (for qPCR assays) are indicated in bold.

Primers and Probes	Organism	Gene	Sequence (5′–3′)	Reference
Bor2F	*Borrelia burgdorferi*	oppA1	GAA GCG ACT ATT ACT CAT C	[27]
Bor2R			GGC TTT TCT AAT TTT AAC GTT	
Bor2-Bb-P			**Red640**-TTC AAT ACA CAC ATC AAA CCA C-**FL**	
GlpQ-F	*Borrelia miyamotoi*	GlpQ	TCC AGA ACA TAC CTT AGA AGC	[26]
GlpQ-R			ATC AAA TCT TTC ACT GAG ACT TA	
GlpQ-P			GAC AAT GTT CCT ATT ATA ATG CAC GAC CC-**FL** **Red640**-GAA ATT GAC ACA ACC ACA AAT GTT GCA C-**PH**	
BAB 1	*Babesia* spp.	18S	TTC AAC GAG TTT TTC CTT	[28]
BAB 1-1			TTC ATC GAG TTT TAT CCC T	
BAB 2			CAG TCC GAA TAA TTC ACC	
BAB 3			GTG ATG GGG ATA GAT TAT TGC AA-**FL**	
BAB 4			**Red640**-TAT TAA TCT TGA ACG AGG AAT GCC-**PO**	
Env-1	Powassan virus	Envelope	GAG AAG CTT ACG AGG TGC ACG CAT CTT GA	[29]
Env-2			GTG CTC GAG TGC GCC TAC TGA GCC TTT GTC CCA	

**Table 2:** Pathogen testing of *Ixodes scapularis* ticks harvested from deer in Wisconsin using quantitative PCR assays. County assignment is based on deer harvest location and may not reflect the site of tick acquisition.

Pathogen	No. Positive	Positive (%)	County
*Borrelia burgdorferi*	38/163	23.3%	Columbia Dane Dunn La Crosse Monroe Sauk Unknown
*Borrelia miyamotoi*	7/163	4.29%	Monroe
*Babesia odocoilei*	24/163	14.7%	Dane Monroe Richland Unknown
*Babesia microti*	1/163	0.613%	Monroe
Powassan virus	1/163	0.613%	Unknown

## Data Availability

The data presented in this study are available NCBI GenBank associated with BioProject PRJNA1304582. U.S. Geological. Survey data release, https://doi.org/10.5066/10.5066/P13OPBRU.

## Supplementary Materials

The following supporting information can be downloaded at: https://www.mdpi.com/article/10.3390/microorganisms13112635/s1, Figure S1: Rarefaction curves based on 16S rRNA gene sequencing of tick microbiota. (A) Rarefaction curves of raw dataset. (B) Rarefaction curves of subsampled dataset to 1035 reads. Figure S2: Rarefaction curves based on Internal Transcribed Spacer 2 (ITS2) region sequencing of tick mycobiota. (A) Rarefaction curves of raw dataset. (B) Rarefaction curve of subsampled dataset to 1000 reads. Figure S3: Bar plot of the relative abundances of bacterial families across *Ixodes scapularis* ticks collected from white-tailed deer in the presence of endosymbiont reads. Bar colors correspond to bacterial families as indicated in the legend to the right. Tick infection status is indicated by colored boxes under the x-axis. Figure S4: Bar plot of the relative abundances of fungal families across *Ixodes scapularis* ticks collected from white-tailed deer. Relative abundance of fungal families associated with individual tick samples is arranged based on infection status and pathogen. Bar colors correspond to fungal families as indicated in the legend to the right. Tick infection status is indicated by colored boxes under the x-axis. Low-abundance families that account for less than 10% of sequence reads per sample are combined and categorized as “Other”. Reads from Operational Taxonomic Units (OTUs) that were not assigned to the family level were combined and categorized as “Unclassified”. Figure S5: Heatmap of the relative abundances of the most abundant fungal genera identified across *Ixodes scapularis* ticks. Heatmap showing hierarchical clustering of tick samples based on Euclidean distance of Operational Taxonomic Unit (OTU) relative abundances. The y-axis displays abundant fungal genera across all samples. The x-axis represents individual tick samples. Figure S6: Alpha diversity indices as measured by observed Operational Taxonomic Unit (OTU) richness (Sobs) and Shannon’s diversity (H’) of infected and uninfected *Ixodes scapularis* samples. (A) Observed Operational Taxonomic Unit (OTU) richness (Sobs) across *Borrelia*-positive and uninfected ticks. (B) Shannon’s diversity across (H’) across *Borrelia*-positive and uninfected ticks. Figure S7: Genus-level bacterial associations across *Borrelia*-infected and uninfected ticks using the Semi-Parametric Rank-based approach for INference in Graphical model (SPRING) as an association measure in the presence of endosymbiont reads. (A) Complete network for the uninfected ticks with 236 genera. (B) Complete network for the Borrelia-infected ticks with 236 genera. Figure S8: Network topology metrics of the uninfected tick microbiota across 50 random subsampling iterations. (A) Network topology in the absence of endosymbiont reads. (B) Network topology in the presence of endosymbiont reads. Table S1: Sequence read counts and per-sample distribution following each step of the mothur pipeline for 16S and ITS datasets as listed in separate tabs. Table S2: Amplicon Sequence Variants (ASVs) identified across individual tick samples collected from white-tailed deer hunted during hunter-harvest season in Wisconsin. Table S3: Fungal Operational Taxonomic Units (OTUs) after subsampling to 1000 reads identified in individual ticks collected from white-tailed deer hunted during hunter-harvest season in Wisconsin. Table S4: Overview of metadata associated with the tick samples collected from white-tailed deer in Wisconsin. Text S1. Mothur Pipeline for 16S rRNA Gene Sequence Processing of Tick Microbiota Samples; Text S2. Mothur Pipeline for ITS region Sequence Processing of Tick Mycobiota Samples.
