# Supplementary figures and images for: Infection-Mediated Shifts in the Microbial Communities of Deer-Fed Ixodes scapularis Ticks
Source: Microorganisms. 2025 Nov 20;13(11):2635. doi: 10.3390/microorganisms13112635 (PMC12654589; doi:10.3390/microorganisms13112635)

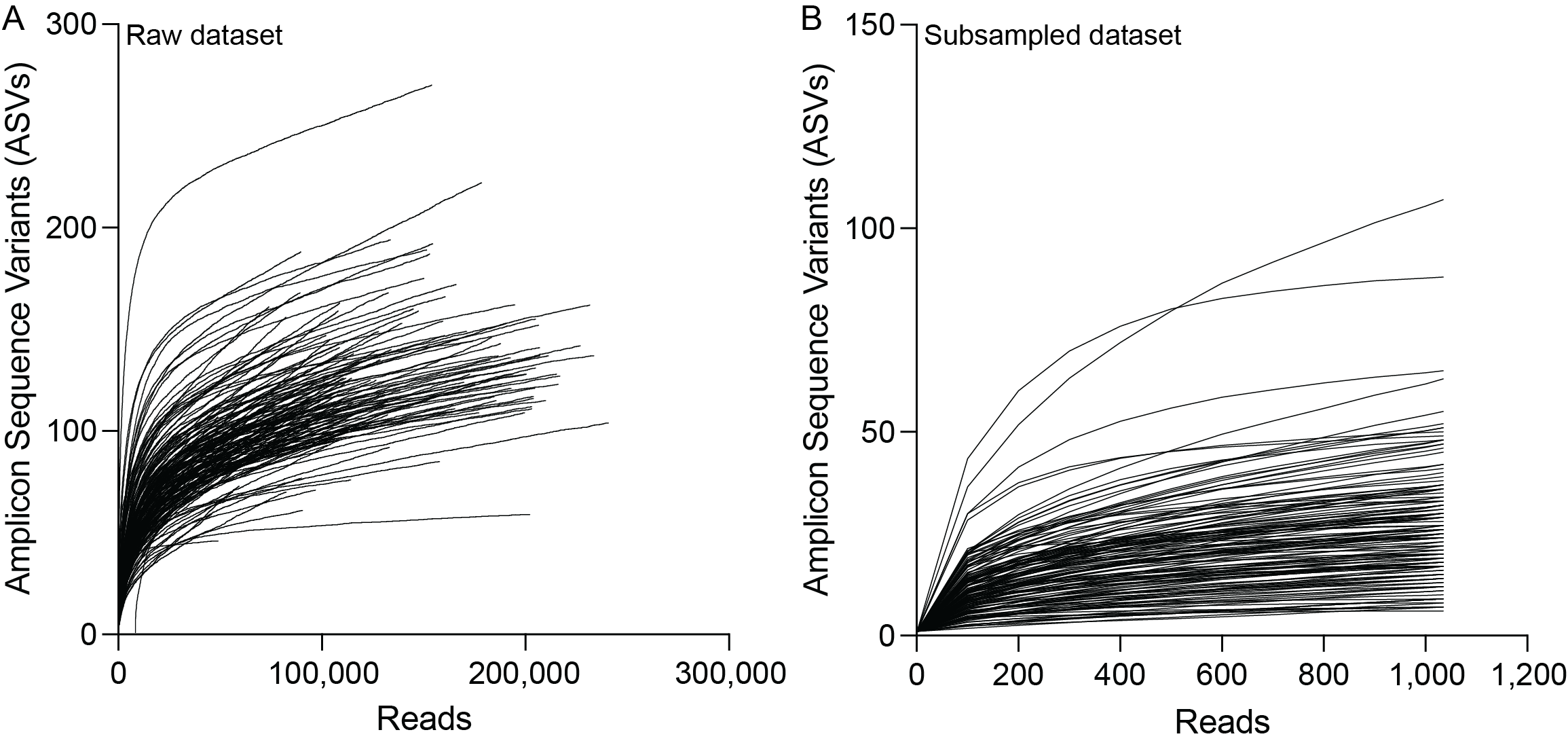

Supplement: Supplementary file 1 [file microorganisms-13-02635-s001.zip › Tawidian2025_FigureS1.png]

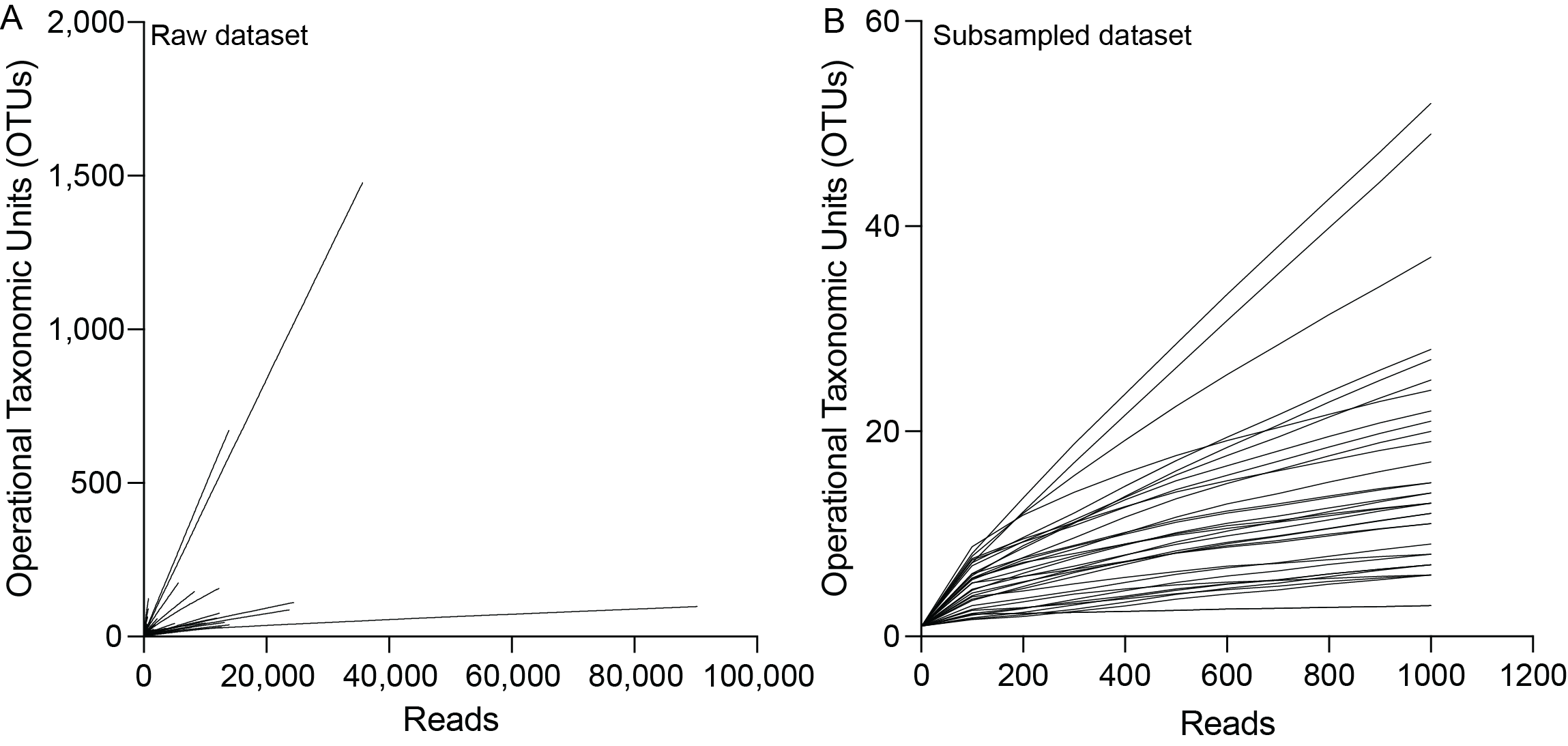

Supplement: Supplementary file 1 [file microorganisms-13-02635-s001.zip › Tawidian2025_FigureS2.png]

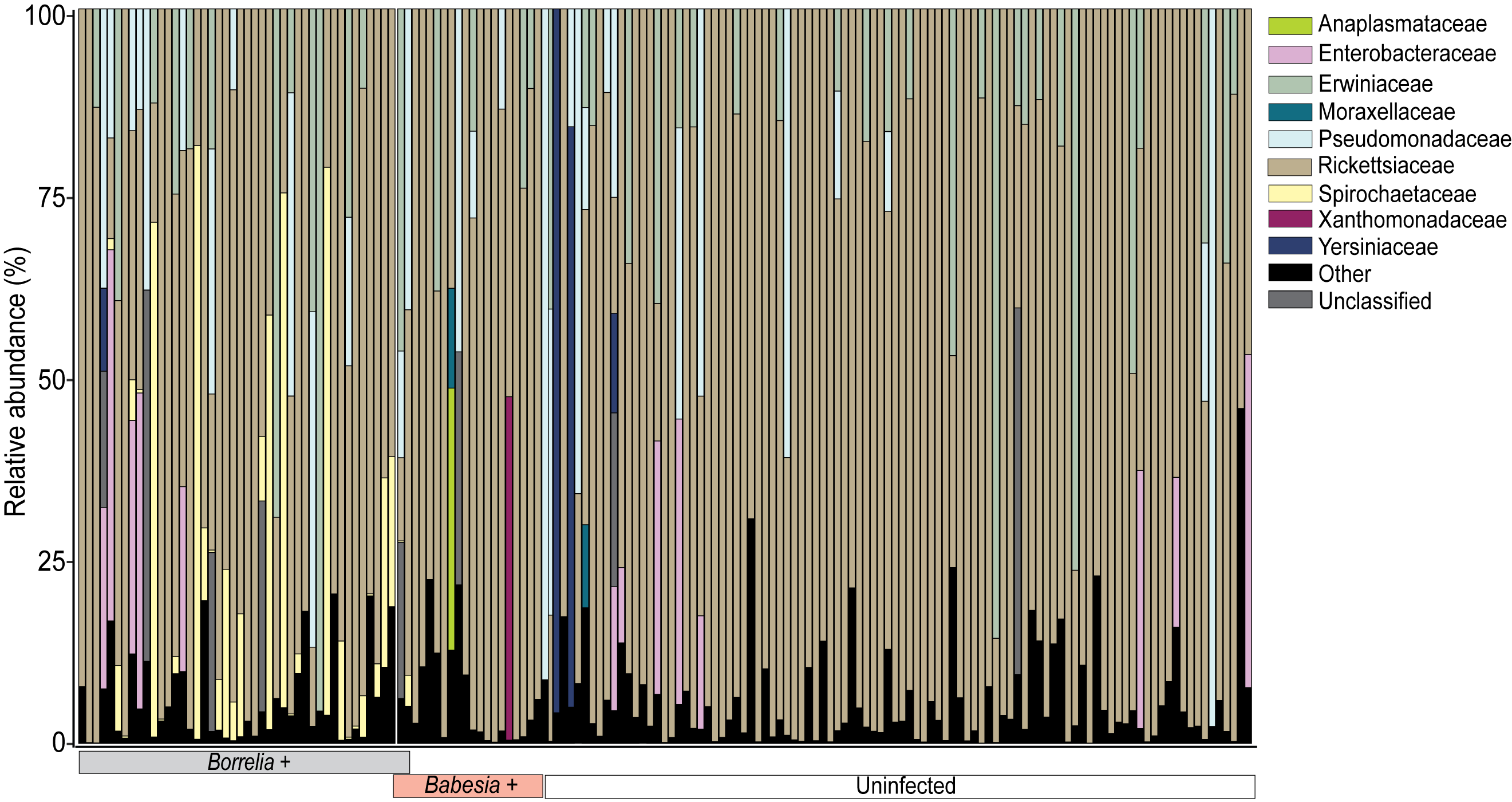

Supplement: Supplementary file 1 [file microorganisms-13-02635-s001.zip › Tawidian2025_FigureS3.png]

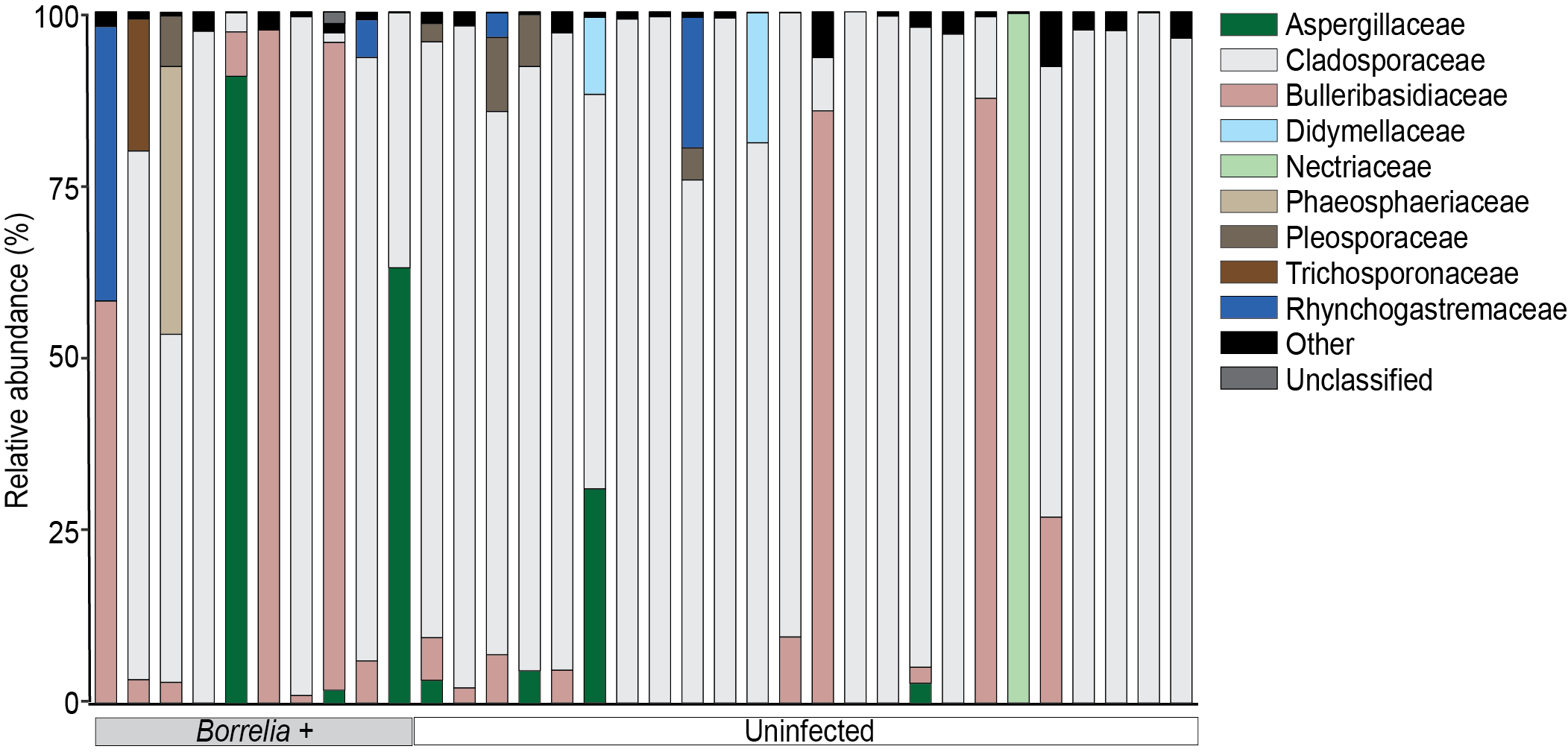

Supplement: Supplementary file 1 [file microorganisms-13-02635-s001.zip › Tawidian2025_FigureS4.png]

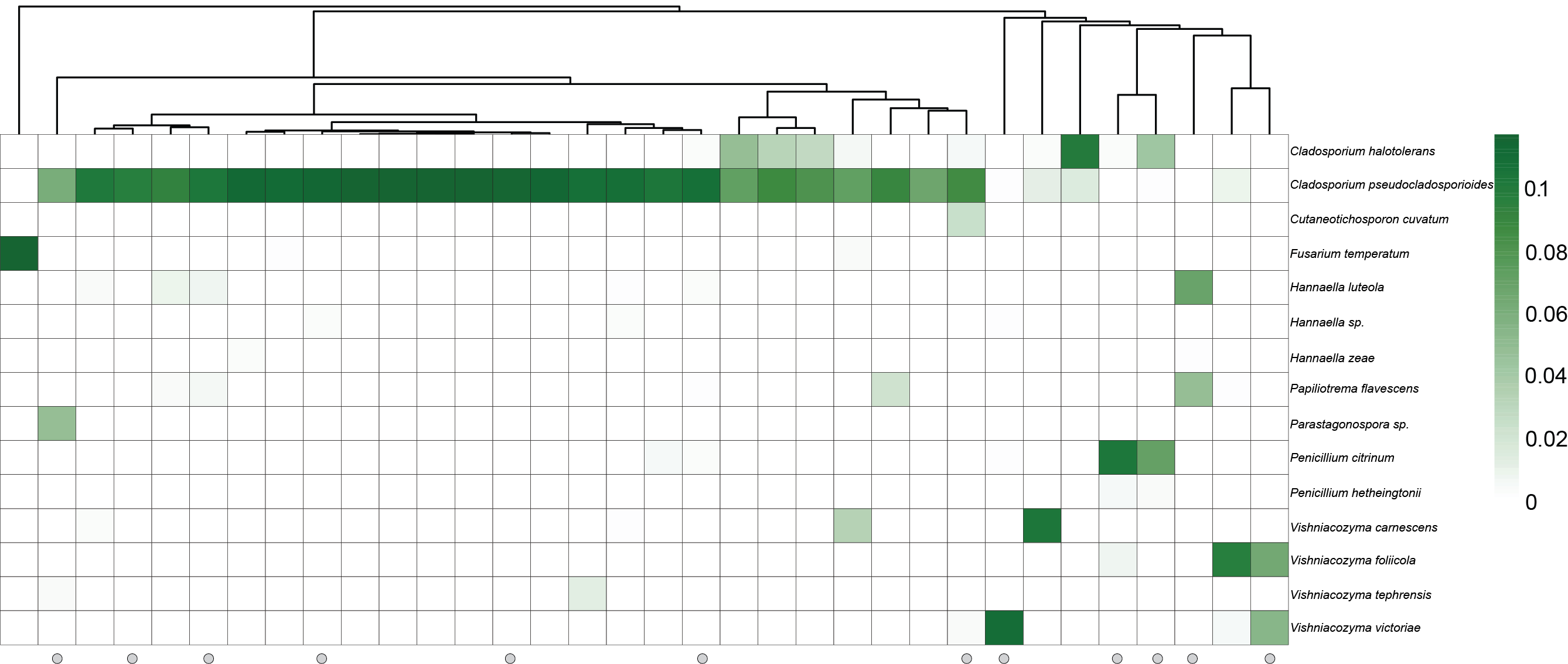

Supplement: Supplementary file 1 [file microorganisms-13-02635-s001.zip › Tawidian2025_FigureS5.png]

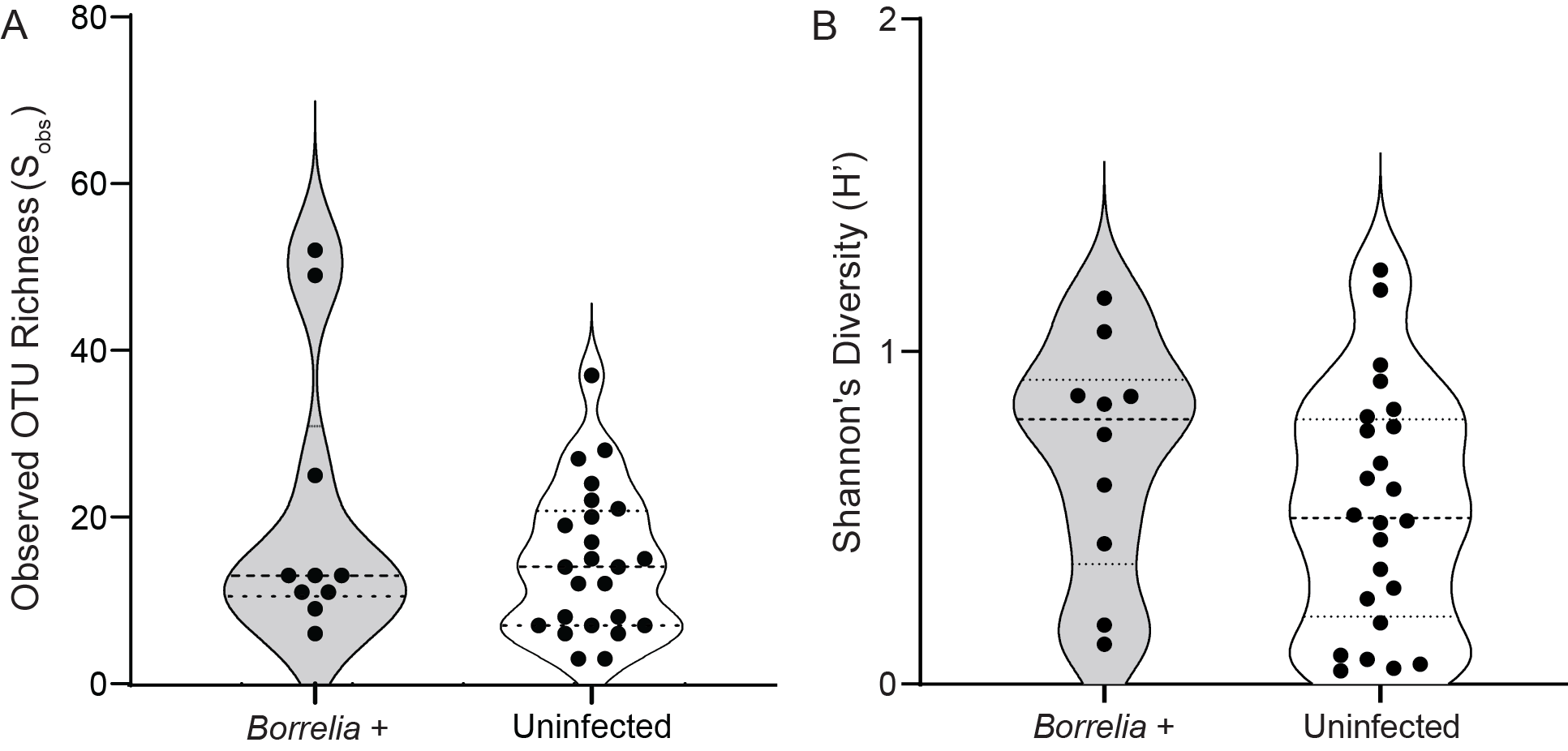

Supplement: Supplementary file 1 [file microorganisms-13-02635-s001.zip › Tawidian2025_FigureS6.png]

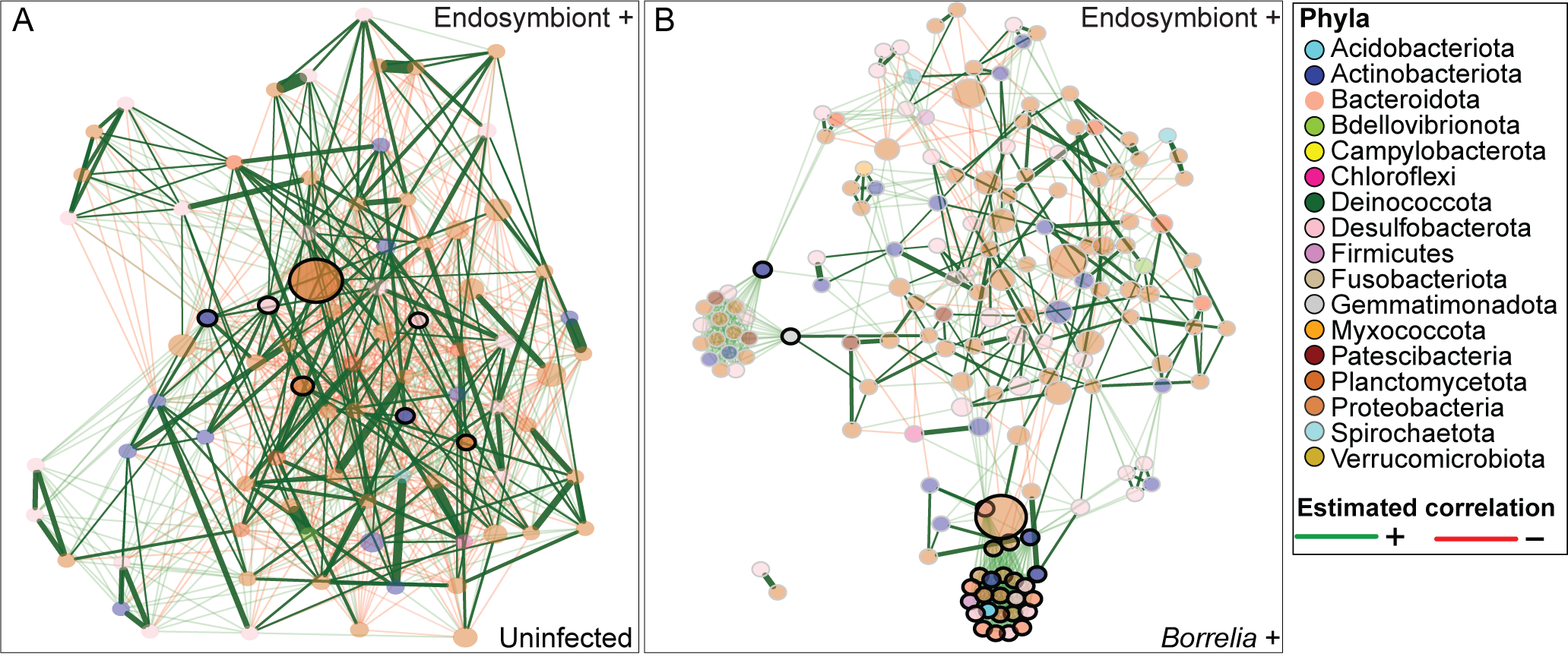

Supplement: Supplementary file 1 [file microorganisms-13-02635-s001.zip › Tawidian2025_FigureS7.png]

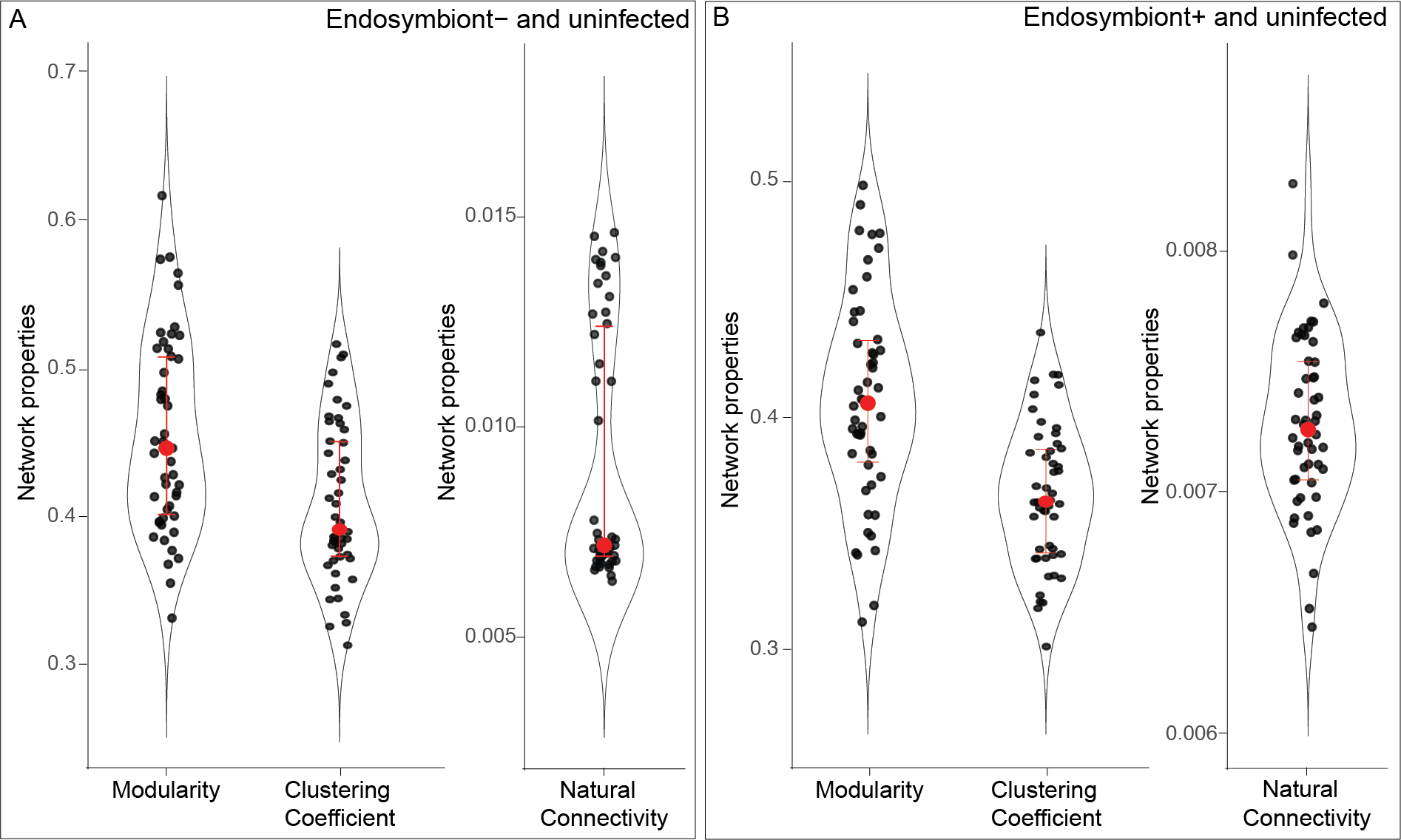

Supplement: Supplementary file 1 [file microorganisms-13-02635-s001.zip › Tawidian2025_FigureS8.png]
